# Unpredictability dictates quality of maternal and newborn care provision in rural Tanzania-A qualitative study of health workers’ perspectives

**DOI:** 10.1186/s12884-017-1230-y

**Published:** 2017-02-06

**Authors:** Ulrika Baker, Farida Hassan, Claudia Hanson, Fatuma Manzi, Tanya Marchant, Stefan Swartling Peterson, Ingrid Hylander

**Affiliations:** 1 Department of Public Health sciences, Global health - Health Systems and Policy Research, Widerströmska huset, Karolinska Institutet, Tomtebodavägen 18 A, 171 77 Stockholm, Sweden; 2 Department of Neurobiology, Care Sciences and Society, Division of Family Medicine, Karolinska Institutet, Nobels allé 12, 141 83 Huddinge, Sweden; 3 Ifakara Health Institute, Plot 463 Kiko Avenue, Mikocheni, Dar es Salaam P.O. Box 78 373, Tanzania; 4 Department for Disease Control, London School of Hygiene and Tropical Medicine (LSHTM), Keppel Street, London, WC1E 7HT UK; 5 International Maternal and Child Health, Uppsala University, Drottninggatan 4, 751 85 Uppsala, Sweden; 6 Makerere School of Public Health, Kampala, Uganda

**Keywords:** Maternal and newborn health, Health workers, District health services, Quality of care, Sub Saharan Africa, Grounded theory

## Abstract

**Background:**

Health workers are the key to realising the potential of improved quality of care for mothers and newborns in the weak health systems of Sub Saharan Africa. Their perspectives are fundamental to understand the effectiveness of existing improvement programs and to identify ways to strengthen future initiatives. The objective of this study was therefore to examine health worker perspectives of the conditions for maternal and newborn care provision and their perceptions of what constitutes good quality of care in rural Tanzanian health facilities.

**Methods:**

In February 2014, we conducted 17 in-depth interviews with different cadres of health workers providing maternal and newborn care in 14 rural health facilities in Tandahimba district, south-eastern Tanzania. These facilities included one district hospital, three health centres and ten dispensaries. Interviews were conducted in Swahili, transcribed verbatim and translated into English. A grounded theory approach was used to guide the analysis, the output of which was one core category, four main categories and several sub-categories.

**Results:**

‘It is like rain’ was identified as the core category, delineating unpredictability as the common denominator for all aspects of maternal and newborn care provision. It implies that conditions such as mothers’ access to and utilisation of health care are unreliable; that availability of resources is uncertain and that health workers have to help and try to balance the situation. Quality of care was perceived to vary as a consequence of these conditions. Health workers stressed the importance of predictability, of ‘things going as intended’, as a sign of good quality care.

**Conclusions:**

Unpredictability emerged as a fundamental condition for maternal and newborn care provision, an important determinant and characteristic of quality in this study. We believe that this finding is also relevant for other areas of care in the same setting and may be an important defining factor of a weak health system. Increasing predictability within health services, and focusing on the experience of health workers within these, should be prioritised in order to achieve better quality of care for mothers and newborns.

**Electronic supplementary material:**

The online version of this article (doi:10.1186/s12884-017-1230-y) contains supplementary material, which is available to authorized users.

## Background

In rural Tanzania, mothers’ utilisation of health care during pregnancy and childbirth is increasing [1–4]. This implies a huge potential to reduce maternal and newborn deaths, but there is a significant ‘quality gap’ in the services provided [5, 6]. While contact coverage of health services is high, effective coverage of the same is low with a small proportion of mothers and newborns receiving those key medical interventions that can prevent or treat complications [1, 7]. This is mirrored in estimates that as many as 83% of all maternal deaths globally could be averted by 2020 through improving quality of care in health facilities alone, without any further increases in institutional delivery [8].

Health workers are the key to realising this potential to increased survival. Without them, mothers and newborns will not receive the care they need [9, 10]. Poor readiness of health facilities in terms of lack of drugs and equipment [11] together with a critical shortage of health workers [12], especially in rural areas, is repeatedly mentioned as causing bottlenecks in health service delivery [13]. Many health workers are also inadequately trained, subject to erratic supervision and work in isolated settings [12]; insufficiently supported for the tasks at hand.

In the face of these challenges, several programs have focused on enhancing the performance of health workers in order to improve quality of care in Tanzania and other low-income settings. These include e.g. Pay-for-Performance (P4P) schemes [14], Standards-Based Management and Recognition (SBM-R) [15], Computer-based Decision Support Systems (CDSSs) [16], the 5-S-approach [17] and collaborative quality improvement using Plan-Do-Study-Act (PDSA) cycles [18]. The theory underpinning these programs is that notwithstanding the challenges of providing care in the context of an under-resourced health system, quality can be improved.

Health workers constitute the core of these programs; both as targets for and implementers of program activities. To understand why and how these programs work, it is therefore vital to investigate the conditions for care provision from a health worker perspective and also, how health workers themselves perceive good quality of care. A few studies have explored health worker motivation and perceptions of working conditions in rural Tanzania [19–22]. Assessments of quality have largely however been analysed from a user perspective, e.g. through exit-interviews on satisfaction [23, 24], or from assessments of health service readiness, e.g. through measuring signal functions for obstetric and newborn care [25, 26].

Our objective was therefore to examine health worker perspectives of the conditions for maternal and newborn care provision and their perceptions of what constitutes good quality of care in rural Tanzanian health facilities.

## Methods

### Study design

This was a qualitative study using a grounded theory approach to guide the analysis [27], based on 17 in-depth interviews with health workers in Tandahimba district, Mtwara region, south-eastern Tanzania. It was nested within a district-wide quality improvement project, called EQUIP (Expanded Quality Management Using Information Power) [28].

### Setting

Tandahimba district is located on the Makonde plateau in rural southern Tanzania, close to the border of Mozambique. The population of about 200,000 people is predominantly subsistence farmers but also engage in cashew nut farming. The road network within the district becomes muddy during the rainy season, making emergency transport difficult.

The district has 32 health facilities providing maternal and child care, all government apart from one faith-based. The district hospital provides comprehensive Emergency Obstetric and Neonatal Care (EmONC) [25] as well as regular childbirth care. The three health centres are supposed to manage basic EmONC and efforts are underway to upgrade their services to include caesarean sections [29]. In reality however, the functions required to provide even basic EmONC are rarely fulfilled, especially in terms of assisted delivery and provision of Magnesium sulphate for eclampsia. The 28 dispensaries provide childbirth care according to their abilities including the administration of Oxytocin when available, both to prevent and manage postpartum haemorrhage. Capacity to provide neonatal resuscitation has recently increased in all facilities through provision of equipment and targeted training [28].

There is a severe shortage of health workers with 52% of clinical posts vacant in the district [29]. Lower cadre health workers, such as medical attendants, often have to take on the responsibilities of higher cadre health workers such as nurses and midwives; an example being to assist women during childbirth (Additional file 1) [29].

Utilisation of health care during pregnancy is high with all mothers attending Antenatal care (ANC) at least once during pregnancy, although only 47% attend at least four times [1, 2]. The proportion of mothers giving birth in health facilities has increased substantially in recent years and was estimated as high as 87% (CI 95%: 77–93) in 2014 [28]. The maternal mortality ratio however remains high at 579 deaths/100,000 live births in the Mtwara region, which is higher than the national MMR average of 432 [30]. Neonatal mortality in was estimated at around 31 per 1000 in 2013 [2].

### Data collection

Interviews with an open sample of 17 health workers providing maternal and newborn care were conducted in 14 health facilities between the 4th and 14th of February, 2014 (Table 1). Respondents were selected purposively, to represent different cadres of health workers and levels of health facilities, with the aim to achieve variation in the data (Table 1).

**Table 1:** Characteristics of health workers interviewed

Cadre of health worker	Interviewed total	Hospital	Health centre	Dispensary
Medical attendant	3		1	2
Nurse/midwife	9	2	3	4
Clinical Officer	4			4
Assistant Medical Officer	1	1		
TOTAL	17	3	4	10

Permission to conduct the interviews was obtained from the District Medical Officer in Tandahimba district. Respondents were contacted in advance, by telephone where possible, else by going to see them in the health facility. They were asked if they would be willing to participate and when a suitable time for the interview would be. At the time of the interview, informed written consent was then sought from all respondents. An information sheet about the study was provided and read out by one of the interviewers. Respondents were informed that they could refuse to participate, withdraw or stop the interview without having to state any reason and without any consequences. All respondents chose to participate. Interviews were held in a private area of the health facility. Only the respondent and interviewers were present. Interruptions to allow respondents to attend to their patients were made and for this reason, some interviews took place over the course of a few hours. The median effective interview time was 1 h 12 min (range 1 h 3 min to max 2 h 7 min).

Interviews were co-conducted in Swahili by the first (UB) and second (FH) authors. UB is a Swedish medical doctor with 2 years’ experience of working as a clinician and program manager in rural Tanzania. FH is a Tanzanian social scientist with experience from several qualitative studies in southern Tanzania. Both are fluent Swahili-speakers and minimal translation into English by FH to UB was done during the interviews. Neither UB nor FH were known to the respondents beforehand. The interview guide was semi-structured and developed in collaboration between all authors of the study. It was adapted after the first interviews as some questions did not yield sufficient response and to reflect new ideas emerging during the data collection (see Additional file 2). While the number of interviews was pre-determined, it was felt that saturation in the interview material was reached before the last few interviews were conducted as no or little new information emerged. No repeat interviews with respondents were done.

Interviews were audio recorded and transcribed verbatim. Subsequent translation into English was conducted with careful attention to quality to ensure preservation of the original meaning. Extensive field-notes were taken during and at the end of each interview. Transcripts were not shared with respondents.

### Data analysis

Data analysis was conducted using Microsoft Word 2010. It was led by the first author, UB, with frequent and substantial input by the senior author, IH. A grounded theory approach was used for the analysis, the end result of which was a core category [27]. Transcripts and field-notes were initially read and re-read to capture the whole. Transcripts were initially coded inductively, using so called open coding close to the text to capture ‘what was going on’ [31]. Similar codes were categorised into higher order categories. After analysing half of the transcripts, a code list was prepared to harmonise emergent codes and categories, agreed upon through discussions between UB and IH. Subsequent transcripts were coded applying these new codes and categories where possible, using focused coding. Theoretical coding was applied early on using the conditional matrix by Corbin and Strauss [31], focusing on the conditions in which health workers provide care and the consequences these have for care provision. As the analysis progressed, theoretical coding continued using the concepts of mothers’ utilisation of care, the availability of resources in health facilities and the actions taken by health workers, i.e. clinical practice, as it became apparent that the data fitted well into these categories. Main categories contained health worker perceptions of the conditions for care provision and their perspectives of what constitutes good quality care. All main categories were linked to the core category. As the core category emerged, interviews were also reread to find more examples of the core category and to saturate the description of the links between the core category and the main categories. Results were not checked by the respondents.

## Results

**Fig. 1:**
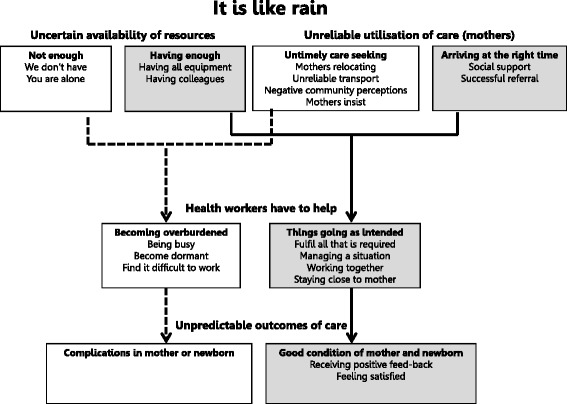
Results framework. ‘It is like rain’ is the core category. The four main categories are outlined in bold with sub-categories contained within the boxes below. Sub-categories in *white* boxes are the ones perceived to challenge quality of care while those in *grey* boxes are perceived to represent conditions for or signs of good quality care. Codes within sub-categories, where applicable, are listed within the boxes. Arrows between boxes illustrate relationships between categories: dashed lines emanate from *white* boxes and full lines from grey

The result of our analysis is a model (Fig. 1) illustrating the core category ‘It is like rain’ with its four main categories: ‘Uncertain availability of resources’, ‘Unreliable utilisation of care (mothers)’, ‘Health workers have to help’ and ‘Unpredictable outcomes of care’. Each main category contains two sub-categories, one of which contains conditions that challenge care provision or perceptions of poor quality care. The other sub-category contains enabling conditions for care provision or perceptions of good quality of care. The results follow the flow in Fig. 1 and are outlined in detail below.

### It is like rain

The notion of uncertainty, represented by the expression ‘it is like rain’, was identified as the core category. Its essence is the inherent unpredictability of the conditions underlying maternal and newborn care provision, as experienced by health workers. One cannot be certain that mothers will come at the right time or that transport will be available. One cannot depend on a regular supply of sufficient drugs or functioning equipment. Colleagues may or may not be available to help manage an unforeseeable work load or a difficult case.

‘It is like rain’ also represents a sense of being out of control; that circumstances are dictated from higher levels of the system while as a health worker, one is left with no choice but to handle the situation on the ground. It is closely linked to how health workers identify good quality of care. When things go as intended, when circumstances are predictable and the system reliable, care provision is more likely perceived to be successful.

### Unreliable utilisation of care (mothers)

Mothers’ utilisation of health services is determined by their access to those services, and is a prerequisite for the provision of timely and appropriate care. Health workers perceived mothers’ utilisation of care as unreliable and determined by factors largely outside their control. Untimely care seeking practices were perceived to affect care provision negatively, while mothers ‘arriving at the right time’ was experienced as an important factor for good quality care provision.

### Untimely care seeking

#### Mothers relocating

Around the time of delivery, some mothers would move to stay close to their parents. Many families would also relocate during the farming season, causing mothers not to be able to visit their usual health facility. This was perceived to disrupt continuity of care and result in an unpredictable work load.

*[…] sometimes people are many because there are people coming here for farming activities, so you may get even ten people for Mtwara […]* (Health worker I5)

### Unreliable transport

Unreliable transport for referral was indicated as the biggest problem for care provision by several health workers. While there was one district ambulance and a few tricycles with side-cars for transporting mothers, these could not be used consistently due to other engagements, unreliable fuel supply, punctures or absence of a driver. Moreover, drivers would sometimes be reluctant to transport a mother who was in a critical condition.

*I was in the health facility where the driver came and he was shocked with the blood all over the place […] We asked him to take the patient to the hospital but he refused […] he was afraid to take her.* (Health worker I5)

### Negative community perceptions

Community perceptions were perceived to influence mothers’ utilisation of care. Commonly held beliefs about when to seek health care, reliance on traditional healers, and negative attitudes towards health workers and health facilities were mentioned as negative influences.

*They see us as people who are there to provide bad service to them […]. They don’t perceive the hospital to be a friendly place.* (Health worker I16)

### Mothers insist

Mothers at high risk of developing complications would be advised to go straight to hospital at the time of delivery. Those who had given birth many times before would however often insist on delivering in the dispensary anyway.

*You can find that many don’t follow what we say.* (Health worker I11)

Health workers similarly perceived that it was difficult to motivate mothers who felt healthy to attend all scheduled postpartum and postnatal care visits.

*We normally advise mothers to come to postnatal care for examination. That’s where we get problems: if a woman thinks she is ok, she doesn’t come.* (Health worker I15)

Although many mothers would attend antenatal care and arrive in time to deliver in the health facility, some would leave before they had received appropriate care such as being tested in ANC. It was common that mothers asked to leave before having been observed for the required 48 h postpartum.

*They [mothers] often stay for 24 h only […]. They are the ones who are asking to leave; they say that they don’t have money.* (Health worker I8)

### Arriving at the right time

Health workers described mothers’ timely arrival at a health facility as key for adequate management of labour and delivery. When mothers arrived in time, health workers were satisfied.

*Most of them come when the labour pain starts, […], so it is just a reasonable time* (Health worker I13)

### Social support

Mothers would usually arrive with one or more members of their family which health workers viewed positively and essential in case of an emergency. Increasingly, mothers were accompanied by their husbands. This was experienced as a positive development and in contrast with traditional values, where men didn’t see it as their role to be involved during pregnancy and childbirth.

*[…] The fathers [before] had the mentality that once they have impregnated a woman then it is the woman’s responsibility to care for the pregnancy, […] his job is done.* (Health worker I4)

### Successful referral

Successful referral of mothers and newborns to the district hospital mirrored the concern for transport availability and was perceived as an important aspect of good quality care. Several health workers described situations when they had phoned for help and the district vehicle had been able to come or alternative transport had been organised.

*Good luck, we managed to refer the baby [with asphyxia]. […] we succeeded to do that.* (Health worker I13)

### Uncertain availability of resources

Health facilities’ availability of material and human resources determines their capacity to provide care. This was experienced as uncertain by health workers; of frequently not having essential drugs and equipment available and of often being alone. The perceptions of quality mirrored this uncertainty.

### Not enough

#### We don’t have

In many health facilities, essential equipment and infrastructure was either not available, not in working order or not enough for the needs of the facility. This was often mentioned as the biggest challenge in providing maternal and newborn care.

*[…] you were eager to provide a good service as it is supposed to be, but you failed because of tools. […] so we are working in a very difficult environment.* (Health worker I17)

*Like now, we have patients who just delivered. If another patient comes, she will not have a place to sleep [because we only have one bed].* (Health worker I2)

Health workers expressed placing orders of drugs and equipment according to their needs but often receiving an insufficient amount. While lower level health facilities relied on the hospital to have drugs in stock, this wasn’t always the case.

*They [at the lower level health facility] don’t have Oxytocin and therefore decided to send her [to the hospital] but when she arrived there, there was also no Oxytocin – you can imagine how it is confused!* (Health worker I7)

### You are alone

Being alone was expressed as a critical determinant of health workers’ capacity to provide care. This included a lack of colleagues, not having adequate knowledge and not being able to influence one’s working conditions.

Some health workers described working alone as a punishment and expressed the lack of colleagues as influencing their clinical practice negatively. Insufficient knowledge to provide specific aspects of care was experienced by some health workers, e.g. how to monitor labour using a Partograph, deciding what pain relief to administer postpartum or how to administer magnesium sulphate for pre-eclampsia.

*I am always alone here [in the health facility] and they [the district health management] are aware of this situation. I went to ask the nursing officer that did you locate me here as a punishment or what? Eeh it is just like a punishment.* (Heath worker I5)

Health workers experienced inaction from higher levels of the health system when voicing deficiencies in infrastructure, requesting equipment or claiming allowances for working out of hours. This created a sense of not being seen, of being on your own.

*For the problem of supplies, truly we have been requesting Tandahimba [the district health management]. […] we only see that they don’t respond.* (Health worker I5)

### Having enough

#### Having all equipment

Having all drugs and equipment was perceived as fundamental to be able to provide good quality care. This was also consistently mentioned as key to improve health services for mothers and newborns.

*Sometimes you have all equipment and necessary things, so you provide good services.* (Health worker I16)

### Having colleagues

Having colleagues to consult or to assist in difficult situations was perceived as important. Sharing tasks was perceived to improve services and to promote provision of good quality care.

### Health workers have to help

Mothers’ unpredictable utilisation of health services along with the uncertain availability of resources affects care provision. Health workers expressed having to help regardless of whether these essential elements were fulfilled and trying to balance resources to continue to provide care.

*I am medical attendant, but because of the problem [shortage of staff], I am also doing deliveries. I can’t tell a patient that the midwife is not around so it is impossible. I must receive the patient.* (Health worker I14)

*[…] we were trying to balance the situation [of lack of Oxytocin] that you can provide [Oxytocin] to one [mother] whom you think is in need and monitor others.* (Health worker I4)

Having to help and balance would sometimes lead to health workers becoming overburdened, but sometimes, of things going as intended.

### Becoming overburdened

Health workers frequently felt overburdened and found it hard to manage due to the lack of resources and colleagues. This was experienced as affecting their performance negatively.

### Being busy

Being the sole provider of maternal and newborn care in a health facility sometimes resulted in competing demands. This was perceived to result in untimely delivery of care.

*You cannot serve [mothers] in time if you are just alone […]* (Health worker I9)

### Become dormant

Health workers who bore the sole responsibility for maternal and newborn care in a facility described never being able to rest; having to be prepared to work whenever a mother needed assistance, to remain in the facility at night and not being able to fulfil family commitments.

*I was alone in the facility […] for a year and a half. […] I didn’t have Saturdays, Sundays, even holidays. Except for the week-ends if there was no patient, I would rest.* (Health worker I6)

Being alone in a work place was also perceived to influence clinical practice negatively in terms of not having somebody to discuss with.

*[In a previous workplace] we were able to share ideas and discuss how to do things, but if you are here alone, you become dormant.* (Health worker I5)

### Finding it difficult to work

Trying to balance care provision by compromising certain aspects of care was often done, e.g. in terms of maintaining adequate hygiene in the health facility. This was sometimes experienced as a threat to one’s own health and while compromises in care provision would be found, these were sometimes experienced as upsetting.

*To sterilise [when chlorine is not available]*, *after delivery you just wash with normal water then you boil them [equipment]. I’m not accepting this method, but it is our circumstance. What will we do? I don’t accept it at all.* (Health worker I13)

### Things going as intended

When care provision was predictable, when things were going as intended, health workers felt that good quality care could be provided.

*If you attend a woman according to your estimation, you feel happy and proud.* (Health worker I7)

### Fulfilling all that is required

To be able to provide all the services that a mother is supposed to receive was expressed as a sign of good quality.

*The things I was supposed to provide to the mother who was delivered, I provided […]. That is why I feel that I have performed well.* (Health worker I10)

### Managing a situation

Even when complications occurred, health workers expressed satisfaction when they had managed to solve the problem and provide care despite the difficult situation.

*[…] So we had to stitch that tear. She was also bleeding a bit; we had to give her normal saline. Now she is a bit better, she says she is fine. […] So I am very happy my patient has improved.* (Health worker I3)

### Working together

Working together was mentioned as a sign of good clinical practice and health workers described critical situations where they would leave other duties to help each other.

*All together when an expectant mother emerges, nobody isolates herself. All together we stop other duties to concentrate on that case until we make sure that the case has been properly attended.* (Health worker I11)

### Staying close to mother

Health workers expressed staying close to a mother as a sign of quality; physically, to be able to monitor and follow-up, but also emotionally in terms of being kind and respectful. Health workers sometimes accompanied a referred mother to the hospital, or went there afterwards for information about her outcome.

*I mean after delivery, she was bleeding excessively and she was getting weaker. Therefore I took her with me, because her spouse had a car so we left together to Tandahimba. I dealt with her until blood transfusion started; that is when I left.* (Health worker I4)

### Unpredictable outcomes of care

Health workers described varying outcomes of care. Some had experienced situations when a mother or newborn had developed serious complications and even died. More frequent were the examples of normal deliveries, free of complications.

### Complications in mother or newborn

Complications such as severe illness or death of a mother or newborn were experienced as deeply upsetting.

*I tried to wipe the baby but the baby didn’t cry at all and then the baby died. Although the baby was abnormal I felt bad, because she [the mother] carried the baby for 9 months and she got nothing - I felt bad.* (Health worker I6)

Health workers also described situations where a mother or newborn had suddenly deteriorated although at first everything had seemed fine. Assisting a mother or baby in such circumstances was experienced as stressful.

*I was alone here when I attended her on delivery. The placenta came out well but suddenly she started bleeding severely. After providing the medicine to control bleeding, I struggled to call them [the district ambulance].* (Health worker I14)

*After severe bleeding, she became unconscious and I decided to put a drip. […] To be honest, I trembled. I was worried, but through experience I put a drip and it was successful.* (Health worker I6)

### Good condition of mother and newborn

Observing a mother and baby to be in a good condition was mentioned as an important sign of having provided good quality care. An active and crying baby who managed to breastfeed was especially appreciated.

*What made me think I had provided good service is the safety of both the baby and its mother; they left this place in a good condition.* (Health worker I11)

### Receiving positive feed-back

Positive feed-back, from mothers, the community or colleagues, was also described as a way of knowing that the services provided had been of good quality. Mothers expressing gratitude was commonly expressed as a sign of quality.

*It is quite certain that when somebody appreciates my service by saying thank you, she only says so when she is satisfied or by asking me when she has to return or when she comes back again whenever she gets problems.* (Health worker I9)

Another sign perceived to indicate that quality of care was provided in a facility was that mothers would come to deliver from villages outside the immediate catchment area.

### Feeling satisfied

A feeling of having done one’s best, of personal satisfaction, was repeatedly expressed as an indicator of having provided good quality care.

*I am satisfied with the service I provided because I had all equipment. […] The good thing was that gloves, Oxytocin and syringes were available so I used them.* (Health worker I5)

## Discussion

Unpredictability was found to be the common denominator for all aspects of maternal and newborn care provision as experienced by health workers. ‘It is like rain’ implies irregular conditions, outside of health workers’ control, including mothers’ access to and utilisation of care, the availability of drugs and equipment and the presence of colleagues in the health facility. This unpredictability does not however imply that care provision is perceived to be of consistently poor quality. Rather, quality of care fluctuates as health workers try to balance available resources with their feelings of being overburdened and their desire for things to go as intended with the outcome being a good condition of mother and newborn, perceived as a sign of good quality care.

The main categories of our findings reflect different aspects of unpredictability. Mothers’ relocating around the time of birth was perceived as one reason for their unreliable access to care. It has previously been associated with low levels of institutional delivery in nomadic pastoralist populations in northern Tanzania [32] but not been explored in other settings. Interestingly, it was perceived to affect both mothers’ ability to seek care and cause an unpredictable work load for health workers.

The uncertain availability of transport, especially in the case of referral, was frequently described as limiting access to care. A recent study from southern Tanzania found that maternal mortality from direct obstetric causes, such as haemorrhage and eclampsia, was nearly four times higher in women living far away from a hospital [33], reinforcing the importance of a reliable referral transport service. Successful referral was equally described as an important aspect of quality care by many health workers.

Tensions between mothers’ and health workers’ assessment of risk were perceived to result in unpredictable care utilisation. This was true especially for multiparous women who were more likely to insist to deliver at home or to deliver in a dispensary rather than the hospital. Similar findings were reported from the same area of Tanzania in a study from the year 2000 [34] and also from western Tanzania, where multiparous women were 30% less likely to deliver in a health facility than nulliparous women [35]. The opposite has been shown for women in their first pregnancy or for women with previous Caesarean sections [36].

Health workers put much emphasis on the uncertain availability of resources and its consequences for care delivery, mirroring findings from a recent study of health service bottlenecks in Tandahimba district [1]. The unreliability of drugs and medical supplies for maternal care has also been observed in other studies from Tanzania as well as Uganda [13, 20].

Health workers expressed feeling alone in the midst of these unpredictable conditions; of not being able to affect much of what determines their ability to provide care. Previous studies from Tanzania have shown similar results, of health workers feeling abandoned and unsupported by the system [21, 37]. Being forced to balance the uncertain availability of resources and mothers’ unpredictable utilisation of health services; often having to help with the knowledge that they weren’t equipped to do so, was expressed by many health workers. This was perceived as demotivating; also corroborating other study findings from the same area of Tanzania [22, 37]. At the same time, managing a difficult situation was experienced as satisfying and expressed as a sign of quality.

These results resonate with research on the “demand-control” model, originally developed by Karasek and Theorell [38]. This model pose that high demands in combination with feeling a lack of control are the most important stressors in the work environment, but that these can be compensated through support from supervisors and co-workers [38, 39]. Similarly, a recent study showed that high job strain and few days off was related to depressive symptoms and burnout but that support from co-workers had a buffering effect [40]. In our study, the core category ‘it is like rain’ clearly describes the out of control aspect and health workers expressed having to help regardless of their capacity to do so. Similarly, the importance of colleagues was emphasised.

Overall, health workers’ perceptions of quality spanned the three dimensions of good quality care as being effective, safe and a good experience for the patient [41]. To ‘fulfil all that is required’, to observe a ‘good condition of mother and newborn’ and ‘feeling satisfied’ can all be seen as health worker perceptions of effective care. Safe care was expressed as ‘managing a situation’ and ‘working together’. ‘Staying close to mother’ can be interpreted as an expression of safety, to monitor closely; and also of health workers understanding the importance of the mother’s experience. ‘Receiving positive feed-back’ was expressed as important and reliable indication that a mother was satisfied with the health services.

### Policy implications

Our findings are a reminder that without appropriate support for health workers; such as a reliable supply of drugs, functioning equipment, colleagues to share the work with and regular supportive supervision from the district or higher level health facilities; little can be achieved in terms of improving quality of care for mothers and newborns. This goes back to the quality ethos for family planning services: “clients’ rights and providers’ needs” [42].

Our findings also point to a need to consider the influences that programs aimed at improving care may have on unpredictability within health services, whether positive or negative. Adding predictability to the dimensions of quality of care and to include this in processes of planning, improving and evaluating health programs could be a way forward. Acknowledging predictability as an important outcome in its own right could help to refocus improvement initiatives around those changes that can be supported reliably and sustained over time. Centralisation of obstetric care, although controversial in terms of its negative impact on access, is likely to have a positive impact on predictability and has been suggested as one way to improve quality of maternal and newborn care in low-income settings [5]. Another example would be to draw fewer conclusions from cross-sectional assessments of health facility readiness and more from fluctuations in the availability of resources over time; recognising that this approach would demand concurrent strengthening of HMIS data and increased funding or redesign of standard approaches to enable repeat surveys. Incorporating alternative options and contingency plans for when conditions for care provision change could be important when creating strategies and guidelines for the implementation of health programs.

### Methodological considerations

This study included health workers of different cadres from a large proportion of facilities within Tandahimba district and as such achieved good variation in the data. Interviews were co-conducted by a social scientist and a physician, both with experience of working in rural Tanzania, allowing for complementary perspectives.

One limitation of our study was that theoretical sampling according to grounded theory methodology wasn’t conducted. However, the first open sample yielded answers to several of the questions that were raised during the analyses. Also, interview questions were revised based on preliminary analyses of the first interviews and the coding followed grounded theory praxis.

Another consideration is the extent to which health workers felt able to share their full experiences with the interviewers. Health workers rarely expressed that they had ever provided sub-optimal care, despite their detailed descriptions of the unpredictable and often challenging conditions for care provision. This finding may be due to social desirability bias, of health workers wanting to make a good impression.

## Conclusions

In the context of weak health systems, improving quality of care is challenging and cannot be done without the engagement of health workers. For health programs to be effective, they need to tackle the inherent unpredictability dictating both conditions for and quality of care provision. We argue that unpredictability may indeed be an important definer of a weak health system and that this finding can be extended beyond maternal and newborn care. Increasing predictability within health services, and focusing on the experience of health workers within these, should be prioritised in order to achieve better quality of care for mothers and newborns.

## Additional files

Additional file 1: Health worker availability in Tandahimba district in 2011. (DOCX 14 kb)
Additional file 2: Interview guide for health worker interviews in Tandahimba February 2014. (DOCX 27 kb)

## Abbreviations

ANC: Antenatal care
CDSS: Computer based decision support system
EmONC: Emergency obstetric and newborn care
EQUIP: Expanded Quality management using information power
PDSA: Plan-Do-Study-Act
QI: Quality improvement
SBM-R: Standard based management and recognition
