# Supplementary material for: Unpredictability dictates quality of maternal and newborn care provision in rural Tanzania-A qualitative study of health workers’ perspectives
Source: BMC Pregnancy Childbirth. 2017 Feb 6;17:55. doi: 10.1186/s12884-017-1230-y (PMC5294891; doi:10.1186/s12884-017-1230-y)
Supplement: Additional file 1: — Health worker availability in Tandahimba district in 2011. (DOCX 14 kb) [file 12884_2017_1230_MOESM1_ESM.docx]

**Additional file 1**

Health worker availability in Tandahimba district in 2011

| **Cadre of health worker** | **Training** | **Number required**  **in district** | **% of positions filled** |
| --- | --- | --- | --- |
| Medical Officer (MO) | 5 years, Medical school | 5 | 40% |
| Assistant Medical Officer (AMO) | 3 years CO College + 2 years AMO training | 16 | 38% |
| Clinical Officer (CO) | 3 years CO college | 116 | 27% |
| Nursing officer | 3-4 years Nursing school | 30 | 57% |
| Nurse midwife | 3-4 years Nursing + Midwifery school | 137 | 25% |
| Medical attendant | 1 year course | 72 | 124% |
| **Total** | | **376** | **52%** |

Description of cadres, number of staff required according to the CCHP (Comprehensive Council Health Plan) 2011-2012 and the proportion of positions filled in 2011 [[27](#_ENREF_27)].
