# Supplementary material for: Unpredictability dictates quality of maternal and newborn care provision in rural Tanzania-A qualitative study of health workers’ perspectives
Source: BMC Pregnancy Childbirth. 2017 Feb 6;17:55. doi: 10.1186/s12884-017-1230-y (PMC5294891; doi:10.1186/s12884-017-1230-y)
Supplement: Additional file 2: — Interview guide for health worker interviews in Tandahimba February 2014. (DOCX 27 kb) [file 12884_2017_1230_MOESM2_ESM.docx]

**NB:** Questions after bullet point are *prompts,* only use if needed.

**Introduction**

1. **Can you tell us a little bit about yourself; how you decided to become a health worker and how and when you came to work in this health facility?**

*We are interested to learn from your experiences of providing care for mothers and newborns in this facility, what works well and what problems you may have.*

1. **To start with, could you tell us a little bit about your work load during the past week?**
   - Clinics held
   - Did anything special happen?
   - How many deliveries did you attend?
2. **Could you describe what usually happens during a delivery in this health facility?**
   - Who accompanies mothers to the health facility?
   - Routine examinations, interventions/drugs
   - How is the mother and newborn cared for after delivery?
   - Complications – referral. In which situations do you refer a mother or newborn? Can you give an example?
3. **Could you tell us about a recent situation where you felt you were able to give a mother or newborn good care/where everything went well?**
   - How were you able to help her? Did you get assistance?
   - What made you feel that you had provided good care?
   - How do you know if a mother is satisfied with the care she receives?
   - Do you ever get reports after the woman has left the facility? How?
4. **Could you tell us about a recent situation where you felt you were not able to give a mother or newborn all the services that you wanted to give them/a situation where you would have wanted to do more?**
   - In what way were you not able to give the care you wanted? What were the consequences?
   - What do you think were the reason for this?
   - What would you have needed to provide good care in this situation?
5. **Thinking about these two examples; what do you feel are the main problems facing health workers in providing maternal and newborn care in Tandahimba district?**
   - What do you think are the reasons for these problems?
   - How could these problems be reduced? By whom? With what resources?
   - How can individual health workers contribute?
   - If you have worked in a different district or health facility before – did you have the same problems there?
   - If these problems were reduced (eg more health workers, equipment), what care would you be able to provide that you are not providing today?
